# Asymptomatic chronic suppurative cholecystitis and peritonitis mimicking metastasis by ^18^F-FDG PET/CT scan during sigmoid colon cancer surveillance

**DOI:** 10.1259/bjrcr.20210046

**Published:** 2021-07-21

**Authors:** Yanqin Sun, MingMing Yu, DaCheng Li, LingLing Sun, Zhenguang Wang

**Affiliations:** 1 Department of Nuclear Medicine, The Affiliated Hospital of QingDao University, Qingdao, China; 2 Department of Pathology, The Affiliated Hospital of QingDao University, Qingdao, China

## Abstract

The study describes an unusual case that a patient with previous history of adenocarcinoma of sigmoid colon who has developed chronic suppurative cholecystitis and peritonitis was misdiagnosed as metastasis. This case is presented to illustrate the importance of considering benign etiologies that may mimic metastatic disease when interpreting positron emmision tomography (PET)/CT scans.

## Clinical presentation

A 75-year-old male with previous history of adenocarcinoma of sigmoid colon (pT3N1aM0) who had undergone a colectomy and a followed 6 cycles of chemotherapy (unknown dose of gemcitabine, po) presented with gallbladder and peritoneum masses 9 months after the surgery. Patient denied any abdominal pain or fever. The laboratory data including white blood cell and neutrophil cell count remained normal (WBC:4.3 × 10^9^/L;Neu:2.0 × 10^9^/L). The CRP level was elevated,10.75 mg/L (normal range:0–5 mg l^−1^). Tumor marker carcinoembryonic antigen(CEA) was normal,whereas carbohydrate antigen 199 (CA19-9) level was elevated with a number of 121.2 U ml^−1^ (normal range: 0–37 U ml^−1^).

## Investigations

**Figure 1.**
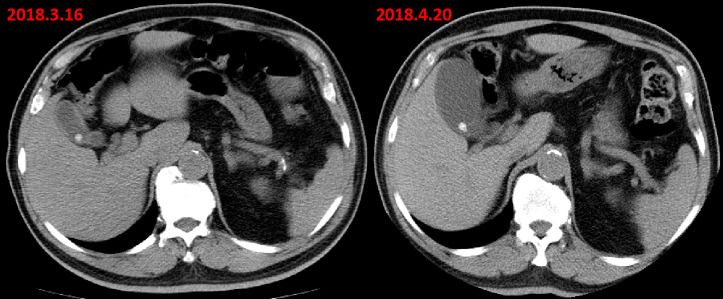
Axial abdominal CT scan (before colon surgery) revealed a slightly uniform thickening of gallbladder wall and gallbladder stones. Post-operative 1 month abdominal CT scan only showed a distended gallbladder and gallbladder stones without thickening of the gallbladder wall. And, there was no abnormalities in the left peritoneum at that time.

**Figure 2.**
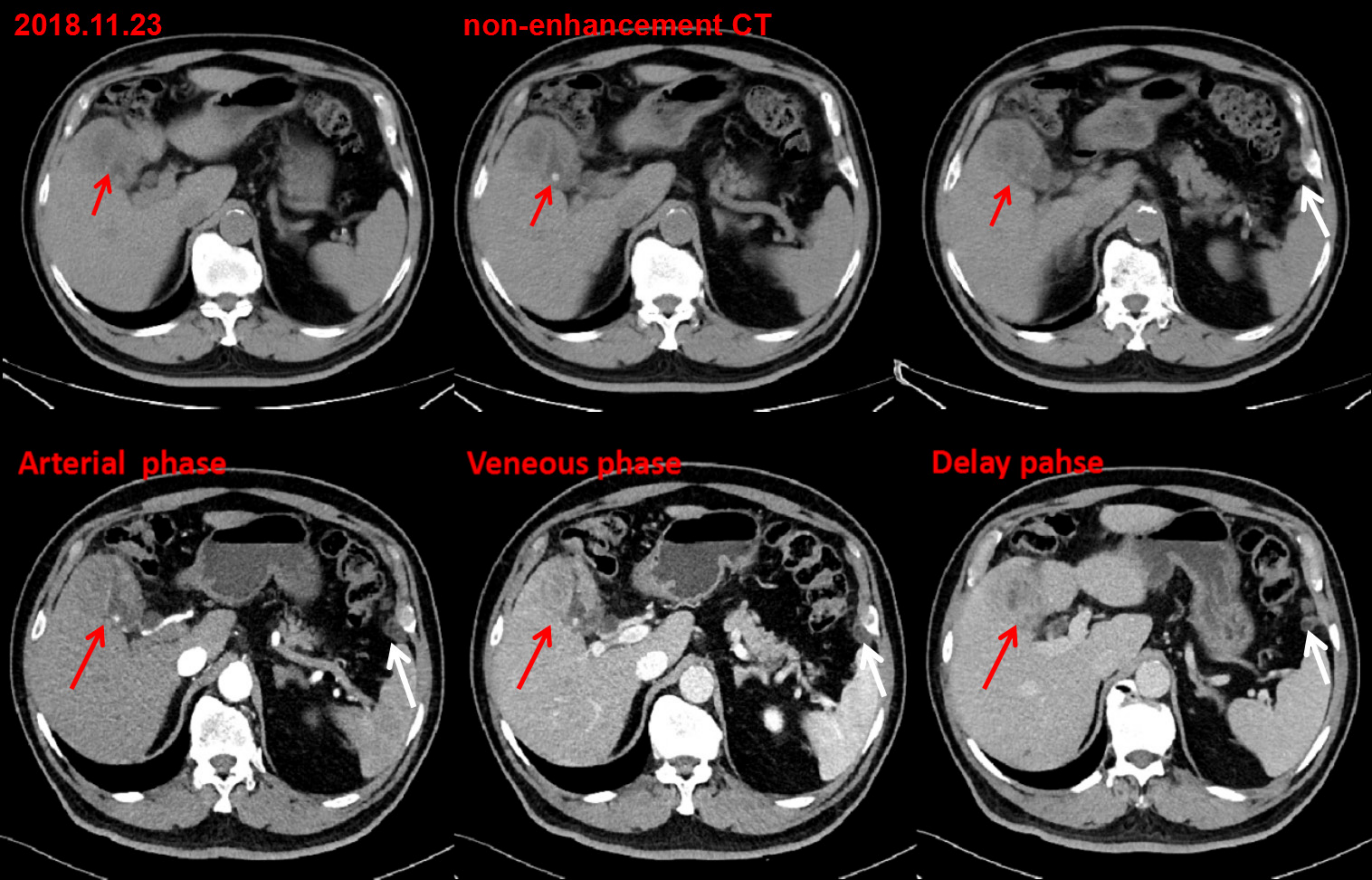
Post-operative 9 month contrast-enhanced CT images detected uneven thickening of gallbladder wall with a progressive enhancement pattern(red arrow), accompanied by gallbladder stones and a rim-like pattern enhancement of left peritoneum(white arrow).

**Figure 3.**
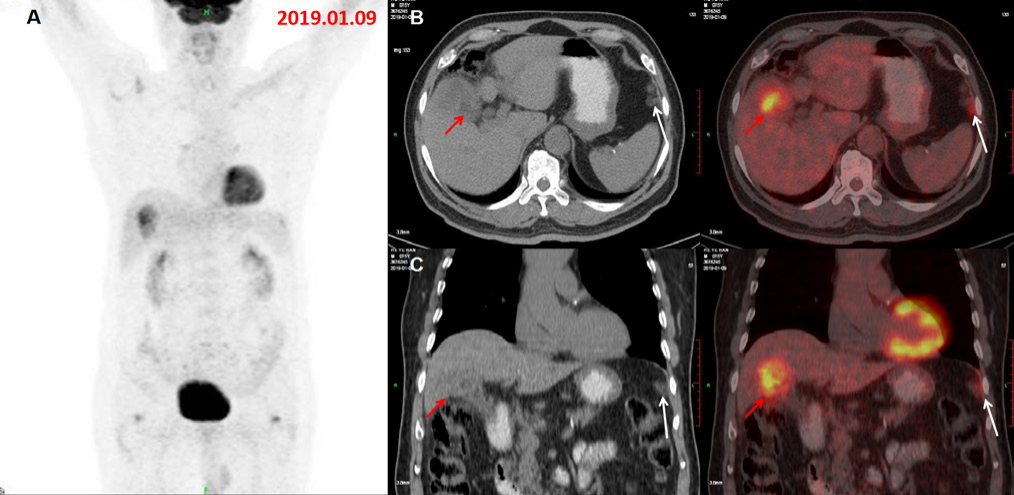
The MIP of the concurrent ^18^F-FDG PET/CT (A) demonstrated two foci with increased metabolic activity in gallbladder bed and left peritoneum. Axial (B) and coronal (C) PET/CT images showed increased FDG accumulation in the irregular thickened wall of the gallbladder, invading liver parenchyma and hepatic flexure of transverse colon, suggesting a malignant gallbladder lesion(red arrow), with SUVmax 5.0 and several nodules and patches on the left peritoneal area(anterior to the spleen) with a slightly increased FDG uptake(SUVmax:3.3), which was not showed on the preoperative PET/CT images, mimicking peritoneal metastasis (white arrow) form colon cancer. FDG, fludeoxuglucose; MIP, maximum intensity projection; PET, positron emmision tomography; SUVmax, maximum standardized uptake value.

**Figure 4.**
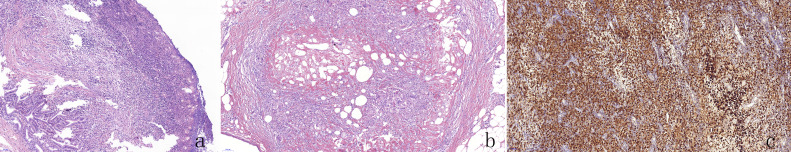
HE staining showed a large number of lymphocytes, plasma cells and neutrophils infiltration, and fibrous tissue hyperplasia, along with the accumulation of foam cells (c) in both gallbladder (a) and left peritoneum (b). No malignant cells were detected (a,b,c,×100) .

The non-contrast abdominal CT scan performed prior colectomy revealed a slightly uniform thickened gallbladder wall and gallbladder stones. Post-operative 1 month abdominal CT scan only showed gallbladder distension and gallstones without gallbladder wall thickening, and there was no abnormalities in the left peritoneum (Figure 1). Post-operative 9 month abdominal contrast-enhanced CT images detected progressive enhancement of the uneven thickened gallbladder wall with gallbladder stones, as well as ring enhancement of the left peritoneum (Figure 2). ^18^F-FDG PET/CT detected highly FDG accumulation in the gallbladder, adjacent liver parenchyma and left peritoneum, most likely suggestive of malignant disease (Figure 3). Based on these imaging features, clinical manifestation and CA19-9 elevation, this patient was highly suspected with metastasis from colorectal cancer. However, post-operative pathology report revealed a chronic suppurative cholecystitis and peritonitis.(Figure 4)

## Differential diagnosis

Metastatic disease is the most common differential diagnosis for these observations of significant FDG accumulation in the gallbladder and peritoneum in a patient with confirmed cancer. It is very difficult to differentiated these two diseases due to the morphological and metabolic characteristics on ^18^F-FDG PET/CT scan, the lack of clinical manifestations related to inflammation, the stage T3 of colorectal cancer, and the elevated serum CA19-9.

Xanthogranulomatous cholecystitis is another disease should be differentiated from, it manifests as uneven gallbladder wall thickening with progressive enhancement, invading the liver and hepatic flexure of the transverse colon.

## Treatment

The patient was treated with laparoscopic cholecystectomy and partial hepatic resection of segment V. Severe intra-abdominal adhesions were found during the operation. A palpable mass was found in the original position of gallbladder bed, adhered with surrounding colic flexure and Segment V of the liver, however the gallbladder could not be exposed. Considering the difficulty of lysis of local adhesion and the impossibility to judge malignant transformation of the gallbladder, so Cholecystectomy + Partial Hepatic Resection (segment V) + Adhersiolysis and Peritoneal Nodules Resection under laparoscope were performed.

## Outcome and follow-up

8 weeks after the cholecystectomy, the serum CA19-9 concentration has reached normal value. There are no current obvious indicators of colon cancer recurrence in this patient.

Patient consent statement has been obtained and signed for this case report to be published.

## Discussion

Chronic cholecystitis is the result of repeated episodes of acute cholecystitis or long-term stimulus from gallbladder stones, and also can be caused by decreased autoimmunity.^1,2^ It has been reported in the literature that patients who has had sigmoid colon cancer and chemotherapy can develop acute episodes of chronic cholecystitis due to their impaired immunity.^3^ Chronic cholecystitis often manifests as even thickened of the gallbladder wall with homogeneous enhancement feature, and significantly high FDG uptake.^4,5^ The molecular basis of ^18^F-FDG uptake by inflammatory tissue is similar to that of tumor tissue, which is monocytes/macrophages in white blood cells (WBCs) take up ^18^F-FDG.^6^ In addition, the special type of chronic cholecystitis, xanthogranulomatous cholecystitis, is an uncommon inflammatory disease of the gallbladder and is characterized by marked proliferative fibrosis, macrophage infiltration, and foam cells invading gallbladder wall and it is difficult to distinguish from gallbladder tumors.^7,8^ This patient had some features similar to xanthogranulomatous cholecystitis, such as uneven thickening of the gallbladder wall with progressive enhancement, invading the liver and right colic flexure. In this case, foam cells can be seen in pathological examination, but no granulomas have formed. Therefore, a diagnosis of chronic suppurative cholecystitis with peritonitis was made. Reviewing diagnosis and treatment process and imaging examination, the reasons for the misdiagnosis are summarized as follows: (1) the lesions significantly took up FDG and invaded the liver and peritoneum. The morphological and metabolic characteristics on PET/CT were similar to those of malignant tumors. Previous studies^9,10^ have also shown that such inflammatory lesions with significant intense FDG uptake are easily misdiagnosed as malignant tumors. (2) Despite a history of chemo-induced immunology damage, there were no clinical signs and symptoms of acute episodes of chronic cholecystitis and peritonitis. (3) With a colon tumor stage pT3N1aM0 and an elevated serum CA19-9, it was easy to infer a serosa-penetration of sigmoid colon cancer and the metastasis to the gallbladder fossa, adjacent liver tissue and peritoneum, which was misdiagnosed as gallbladder tumor. (4) Conventional CT and enhanced scans both suggested a metastatic tumor, which subjectively affect the diagnosis of PET/CT examination. In actual work, atypical or rare cases may lead to misjudgment or misdiagnosis. It is worth noting that CA19-9 can also be elevated during cholecystitis, and attention should be paid to the characteristics of such cases in history, clinical manifestations and various examinations which may lead to important clues for diagnosis in the future.

## Learning points

1. The case presented illustrates patients sigmoid colon cancer can cause chronic cholecystitis due to impaired immunity.
2. It is worth noting that CA19-9 can also be elevated during cholecystitis, not only in patients with malignant tumor.
3. Asymptomatic chronic suppurative cholecystitis and peritonitis mimicking metastasis by ^18^F-FDG PET/CT scan during sigmoid colon cancer surveillance.
